# The effects of warmed carbon dioxide pneumoperitoneum nursing care on core body temperature, acid-base balance, and lymphocytes in older patients with cancer in Korea: a randomized controlled trial

**DOI:** 10.7586/jkbns.25.055

**Published:** 2025-11-24

**Authors:** Hyosun Park, Nayeon Shin, Jeong-Heum Baek

**Affiliations:** 1 Department of Nursing, Naeun Hospital, Incheon, Korea; 2 Nursing Department, CHA Bundang Medical Center, Seognam, Korea; 3 Division of Colon and Rectal Surgery, Department of Surgery, Gil Medical Center, Gachon University College of Medicine, Incheon, Korea

**Keywords:** Nursing care, Monitoring, physiological, Randomized controlled trial

## Abstract

**Purpose:**

This study aimed to examine the effects of warmed carbon dioxide pneumoperitoneum nursing care on core body temperature, acid–base balance, and lymphocyte levels in older patients with cancer undergoing laparoscopic surgery.

**Methods:**

A total of 55 patients with colorectal cancer were prospectively randomized and analyzed, including 29 in the intraperitoneal CO_2_ gas heating group and 26 in the non-heating group. Data were analyzed using the t-test, chi-square test, and repeated-measures analysis of variance.

**Results:**

Significant differences were observed between the experimental and control groups in body temperature (F = 2.47, *p* = .010), acid–base balance (F = 2.39, *p* = .015), and lymphocyte percentage (F = 4.45, *p* = .002) during laparoscopic surgery.

**Conclusion:**

Warmed carbon dioxide pneumoperitoneum was more effective in preventing intraoperative hypothermia induced by general anesthesia than non-heated gas. These findings suggest that this intervention may be beneficial for older patients with cancer undergoing laparoscopic surgery.

## INTRODUCTION

As of 2021, the life expectancy of the Korean population is 83.6 years, and the elderly population aged 65 and above constitutes 16.5% of the total population, numbering 8,537,000 individuals [1]. Cancer has maintained its position as the leading cause of death among the elderly, with cancer patients aged 65 and above showing a continuously increasing trend at a rate of 1,552 cases per 100,000 individuals [2].

Among these cases, the incidence of colorectal cancer ranked 2th in terms of occurrence according to domestic cancer incidence statistics of 2022 [3]. Laparoscopic surgery for colorectal cancer aims to reduce pain and stress after the procedure, promotes rapid recovery, and decreases hospitalization days [4]. As such, its aesthetic benefits and emphasis on immune system stability have led to its increasing preference [5].

Laparoscopic surgery for colorectal cancer is a surgical procedure involving an injection of gas through the trocar into the abdominal cavity, using a camera for visualization, and manipulating instruments to perform the surgery. During surgery, a gradual decrease in body temperature occurs due to the continuous infusion of 21°C CO_2_ gas into the abdominal cavity [6]. Additionally, the elderly have higher likelihoodof experiencing disease complications due to decreased organ function, and a higher likelihood of hypothermia due to decreased thermo regulatory capacity [4]. In particular, intraoperative hypothermia is caused by the use of 15°C inhaled anesthetic gas during surgery and 21°C CO_2_ gas injected into the abdominal cavity during laparoscopic surgery [6]. Intraoperative hypothermia leads to an increase in catecholamine’s, resulting in elevated blood pressure and heart rate, tissue hypoxia, and metabolic acidosis [4].

Moreover, stress-induced cortisol elevation due to stressors such as hypothermia, trauma, and the procedure itself can suppress immune function by interfering with lymphocyte and macrophage activity, resulting in immune suppression due to a decrease in lymphocytes [7,8]. During surgery, CO_2_ gas injected into the abdominal cavity can lead to easy absorption and potentially cause hypercarbia, stimulating the sympathetic nervous system and inducing respiratory acidosis, leading to disturbances in acid-base balance [9]. Warmed CO_2_ gas at 37°C has been reported to cause acidosis by increasing absorption of CO_2_ gas in the body, based on Fick's diffusion law [10]. The utilization of CO_2_ gas warmed to 37°C offers benefits such as reduced peritoneal damage, decreased postoperative pain, and a lower risk of hypothermia [11]. The evidence supporting the use of warmed CO_2_ gas suggests its potential contribution to enhanced recovery and patient safety in laparoscopic surgery. Therefore, investigating the impact of warmed CO_2_ gas at 37°C on acid-base balance during laparoscopic surgery is crucial. Various nursing interventions during surgery for preventing hypothermia include warming the skin, using warmed intravenous fluids, warm humidification of anesthetic gases, and applying warm insufflation of gas [12]. Previous studies have explored the differences between cold and warmed CO_2_ during laparoscopic procedures [11,13]. Research indicates that cold, dry CO_2_ can induce peritoneal damage and increase lymphocyte infiltration, whereas warmed and humidified CO_2_ mitigates injury [11]. CO_2_ gas used at room temperature can cause peritoneal injury and inflammation, primarily attributed to the cold temperature of the gas [13]. Studies examining the effects of warmed CO_2_ on acid-base balance have shown minimal clinical impact on arterial pH and pCO_2_ levels during laparoscopic cholecystectomy procedure [11]. In particular, the elderly has a lower threshold for vascular contractile responses compared to adults, leading to slower recovery to normal body temperature when becoming hypothermic during surgery [14,15], and despite the high risk of hypothermia during laparoscopic surgery, basic nursing interventions for maintaining body temperature have been insufficient. Therefore, nursing intervention studies supplemented by experimental studies on warm insufflation of CO_2_ gas should be conducted and evidence-based and proactive nursing interventions should be provided. It is necessary to confirm the effect of combined skin warming, fluid warming, anesthetic gas warming, and undulating gas warming in elderly laparoscopic surgery subjects with a study that includes objective indicators to determine the total effect on not only body temperature but also acid-base balance and lymphocytes, which are indicators of physiological response, and to confirm the effectiveness of nursing interventions.

## METHODS

### 1. Study design

This is an experimental study with a randomized controlled trial design with a pre-posttest. In order to minimize the selection bias between the experimental and control groups, the prescribed method was established by creating a randomized table with a Microsoft Office 2010 Excel program (Microsoft Co., Redmond, WA, USA).

### 2. Participants

**Figure 1.**
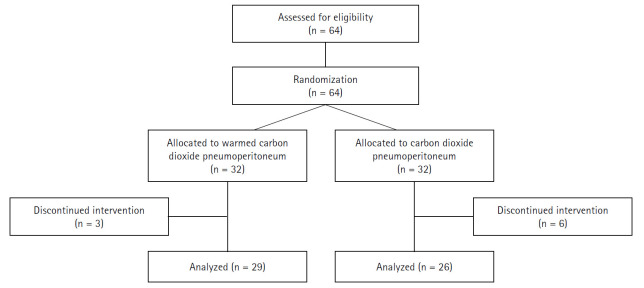
Flow diagram of the process (based on Consolidated Standards of Reporting Trials [CONSORT] statement).

A total of 64 eligible subjects were selected based on pre-defined criteria, using the block blind randomization method. The inclusion criteria for the subjects in this study were as follows: 1) Elderly patients aged 65 years or older, 2) Patients classified as Class Ⅰ or Class Ⅱ according to the American Society of Anesthesiologists Physical Status Classification System [16], undergoing laparoscopic surgery for colorectal cancer with a surgical duration within 3 hours, 3) Patients without preoperative body temperature elevation. Exclusion criteria were as follows: 1) Patients with preoperative hematological issues, 2) Patients who did not receive intraoperative blood transfusion due to the known fact that blood transfusion of 1 pint of blood stored at 4~8℃ [17] reduces body temperature, 3) Patients who did not receive medications (clonidine, phenothiazine, meperidine series) that could affect body temperature during surgery, 4) Patients with a history of chronic obstructive pulmonary disease or heart disease that may cause difficulty in CO_2_ expiration, 5) Patients with thyroid disease that could influence body temperature regulation. The study recruited voluntary patients who met the inclusion criteria and provided informed consent. Recruitment occurred through posting of advertisements in the wards of Gachon Gil Medical Center in Incheon City from October 1 2020, to December 31, 2020. Subjects who expressed interest in participating in the study signed informed consent forms and medical record access forms. The selected subjects were identified based on their eligibility using medical records. Among them, numbers were assigned to the samples that met the subject selection criteria, and random allocation was conducted using an excel program with a random number generation list. The sample size was calculated using the G*Power 3.1.2 program (Heinrich-Heine-Universität Düsseldorf, Düsseldorf, Germany) to maintain the effect size (d) of 0.3, significance level (α) of .05, and power (1-β) of .80 derived from a previous study [18], and the number of subjects was 25 for the experimental group and 25 for the control group, yielding a total sample size of 50 subjects, and a minimum of 32 subjects were selected for each group considering a dropout rate of 20%, resulting in a total of 64 subjects. The 64 enrolled subjects were randomly assigned to 32 experimental and 32 control groups. To reduce the risk of intervention contamination, control group assignment was prioritized, and the experimental group was recruited and delivered the intervention after completing the measurements, and to minimize experimenter effects, a research assistant administered the intervention for warm insufflation of CO_2_ gas, and the principal investigator conducted the pre-survey. One other research assistant conducted the mid-survey and post-survey. Research validity was enhanced by applying a blinding method to the assistant researchers who performed the measurements, not revealing whether they were dealing with the control or experimental group. During the study, three in the experimental group and six patients in the control group and dropped out due to intraoperative blood transfusion. Excluding these dropouts, 29 subjects in the experimental group and 26 subjects in the control group completed the study, with a total of 55 subjects available for analysis (Figure 1).

### 3. Instruments

#### 1) Body temperature

Core body temperature was measured every 15 minutes during surgery by placing an esophageal stethoscope (#4-9552-D, DeRoyal, TN, USA) in the lower third of the esophagus through the nose and connecting it to a monitor. This is based on a previous study [19] that compared the time and magnitude of the largest intraoperative decrease in body temperature in 14 surgical subjects and found that body temperature changed significantly every 10~15 minutes.

#### 2) Acid-base balance

Acid-base balance was assessed by puncturing the radial artery and drawing 1 ml of arterial blood with a heparin-coated 1 ml syringe through a connected monitoring kit (PX 260, Edwards lifesciences, Irvine, CA, USA) and analyzing it with a gas analyzer (Gem premier 3000, Werfen, San Diego, CA, USA) to measure pH, pCO_2_ mmHg, HCO_3_^-^, and base excess. Measurements were taken preoperatively and every hour during surgery for a total of five times. The normal range for each measured variable is pH 7.35 to 7.45, pCO_2_ 35 to 48 mmHg, HCO_3_^-^ 18 to 23 mEq/L, and base excess 0 ± 2 meq/L.

#### 3) Lymphocytes

Lymphocytes were collected in 2 ml of venous blood in anticoagulated ethylene diamine tetra acetic acid tubes, centrifuged by immuno turbid metric assay, and analyzed using an automated hem cytometer (Coulter Ac*T, Brea, CA, USA). Lymphocytes were measured preoperatively, 1 hour postoperatively, 1 day postoperatively, 3 days postoperatively, and 5 days postoperatively at 8:00 am.

#### 4) Warm insufflation of CO_2_ gas

Insufflation of nitrous oxide, carbon dioxide, or air is commonly used to distend the abdominal cavity to secure the surgical field of view and manipulate the contents of the abdominal cavity. Based on previous research showing that warming CO_2_ gas from 21℃ to 37℃ and injected it into the abdominal cavity resulted in a 0.5°C increase in core temperature, this study used the WISAP dedicated cable (Endo-Digi-View 3mlD, WISAP, Brunnthal/Hofolding, Germany) to warm the CO_2_ gas from 21℃ to 37℃.

### 4. Experimental intervention

In this study, in order to prevent contamination between groups, we collected the data of the experimental group after completing the data collection of the control group to increase the validity of the study. To create and maintain the same experimental conditions for the experimental and control groups, the operating room temperature was kept at 22~26℃ and humidity at 30%~40%, and the same operating room was designated to control for variations in the surgical environment. For induction of anesthesia, all patients received intramuscular injection of anticholinergic agents as preoperative medication, 2, 6-diisopropylphenol Rocuronium bromide agents as muscle relaxant intravenously, and after endotracheal intubation, breathing was maintained with a ventilator. The skin was disinfected using room temperature povidone-iodine. Immediately after induction of anesthesia, an esophageal stethoscope was inserted into the lower third of the esophagus, the warm humidification equipment was activated to measure the body temperature using a thermometer on the warming device. For both experimental and control groups, the same method was used before the induction of anesthesia: an electrically circulating water blanket was placed on the patient's back to keep the temperature at 38°C, the intravenous fluids were heated to 41°C with a fluid warmer for intravenous infusion, and the anesthetic gas was heated to 41°C by an electric device (Heated Humidification Circuit A10677, Medimed, Seongnam, Korea). The experimental group was connected to a cable dedicated to insufflation of CO_2_ gas and applied to the insufflation by warming it to 37℃, while the control group applied general CO_2_ gas at 21℃. In addition to the above main variables, data on age, gender, diagnosis, type of surgery, amount of fluid infusion, operating room temperature and humidity, surgical time, and anesthesia time were collected from the study data collection sheet. General and surgery-related characteristics of the study subjects were collected from the patients' medical records.

### 5. Data collection

Data collection for this study was conducted by the principal investigator and three research assistants at Gachon University Hospital in Incheon, Korea. The preoperative data of the study subjects were collected by the researcher on the day before surgery for the experimental and control groups. Data collection from the start of surgery to the end of surgery was performed by one research assistant to ensure continuity of data collection, and blood draws were performed by two other research assistants.

### 6. Data analysis

The collected data was analyzed using IBM SPSS Statistics version 26.0 (IBM Corporation, Armonk, NY, USA). General characteristics and disease-related characteristics of the experimental and control groups were presented as frequencies, percentages, means, and standard deviations. Homogeneity tests between the two groups based on their general and disease-related characteristics were performed using Chi-square test, independent t-test, and Fisher's exact test. The normality of body temperature, acid-base balance, and lymphocytes in the experimental and control groups was assessed using the Shapiro-Wilk normality test. Homogeneity tests for these variables between the experimental and control groups were performed using independent t-test and Mann-Whitney U test. Differences between the experimental and control groups at different time points in terms of body temperature, acid-base balance, and lymphocytes were assessed using independent t-test. Changes in these variables over time within each group (1st, 2nd, and 3rd measurement) were analyzed using Repeated Measures analysis of variance. In cases where sphericity assumptions were violated, Greenhouse-Geisser corrections were applied for the analysis.

### 7. Ethical considerations

The study was carried out after obtaining approval from the institutional review board (IRB) of Gachon University Hospital prior to data collection (IRB No. GFIRB2019143). Subjects undergoing laparoscopic colorectal cancer surgery were given a verbal explanation of the study objectives and procedures the day before surgery and written informed consent was obtained. Before completing the questionnaire, participants read an explanation sheet detailing their right to refuse participation at any time without facing any negative consequences. After completing the questionnaire, placed their responses in a provided envelope before depositing them in a response box.

## RESULTS

### 1. Pre-homogeneity test between experimental and control groups

#### 1) Homogeneity test of general characteristics and disease-related characteristics

General characteristics and disease-related characteristics of the study participants, such as gender, diagnosis, age, surgery type, initial body temperature during anesthesia induction, and surgery duration, were investigated. Chi-square test and t-test were performed to verify the homogeneity between the experimental and control groups for all variables, and the results showed no statistically significant differences, indicating homogeneity between the two groups (Table 1).

#### 2) Homogeneity test of dependent variables in the experimental and control groups

The normality of the dependent variables, including body temperature, acid-base balance, and lymphocytes, was assessed separately for the experimental group (29 participants) and the control group (26 participants) using Kolmogorov-Smirnov and Shapiro-Wilk tests. Body temperature, acid-base balance, and lymphocytes were found to follow a normal distribution. Based on these results, independent t-tests were conducted to verify the homogeneity of body temperature (t = 1.01, *p* = .977), acid-base balance (t = 0.59, *p* = .601), and lymphocytes (t = 1.42, *p* = .201) between the experimental and control groups. The analysis showed no statistically significant differences, indicating homogeneity in these variables between the two groups. It can be concluded that there were no statistically significant differences in the dependent variables between the experimental and control groups before the intervention (Table 2).

#### 3) Body temperature

There will be a difference in intraoperative body temperature between the experimental group with warm insufflation of CO_2_ gas and the control group without warm insufflation of CO_2_ gas. A repeated-measures analysis of variance was conducted to measure the subjects' body temperatures in both groups at three different time points. The analysis did not show a statistically significant difference between the experimental and control groups (F = 0.85, *p* = .359). Additionally, there was a significant difference across different measurement time points (F = 9.49, *p* < .001). The interaction between time and group showed a statistically significant effect (F = 2.47, *p* = .010) (Table 3).

#### 4) Acid-base balance

There will be a difference in intraoperative acid-base balance between the experimental group with warm insufflation of CO_2_ gas and the control group without warm insufflation of CO_2_ gas. A repeated-measures analysis of variance was performed to measure the acid-base balance in both groups at three different time points. The analysis did not reveal a statistically significant difference between the experimental and control groups (F = 0.10, *p* = .751). There was a significant difference across different measurement time points (F = 4.95, *p* <.001). The interaction between time and group showed a statistically significant effect (F = 2.39, *p* = .015) (Table 3).

#### 5) The lymphocytes Count

The lymphocytes count after surgery differs between the experimental group with warm insufflation of CO_2_ gas and the control group without warm insufflation of CO_2_ gas [13]. A repeated-measures analysis was conducted to measure the lymphocyte count in both groups at three different time points. The analysis did not show a statistically significant difference between the experimental and control groups (F = 1.64, *p* = .206). There was a significant difference across different measurement time points (F = 8.49, *p* < .001). The interaction between time and group showed a statistically significant effect (F = 4.45, *p* = .002) (Table 3).

## DISCUSSION

The results of this study demonstrated that insufflation of warmed CO_2_ gas affects body temperature, acid-base balance, and lymphocytes during laparoscopic surgery in the elderly. In this study, the body temperature of the experimental group was measured to be higher than the body temperature of the control group. A temperature change of 0.2°C or more from hypothermia below 36°C is a physiologic change in the human body that has important clinical implications, [11] emphasizing the importance of nursing interventions to prevent hypothermia in laparoscopic surgery patients in this study, the use of 37°C warmed CO_2_ gas resulted in a body temperature increase of 0.21~0.33°C at 30 and 60 minutes after warming compared to insufflation of 21°C CO_2_ gas. The introduction of −90°C compressed CO_2_ liquefied gas into the body, entering the abdomen at a low temperature of 21°C during laparoscopic surgery leads to intraoperative hypothermia [7]. Hypothermia refers to a core body temperature of 36°C or below and in surgical rooms with temperatures between 18°C and 21°C during surgery, the body temperature can reduce to 36°C or below regardless of the patient's body exposure [9]. Furthermore, large amounts of intravenous fluids at room temperature are infused, and after induction of general anesthesia, the use of inhalation anesthetics, muscle relaxants, and antipyretics can cause vasodilation and contribute to intraoperative hypothermia [7]. These results were consistent with a previous study that reported that warming carbon dioxide to 37°C elevates body temperature during laparoscopic surgery [18], but differs from other studies that reported that warming carbon dioxide was not effective in preventing hypothermia in surgical patients [6]. Furthermore, it has been observed in a study that the group undergoing laparoscopic surgery with warm insufflation of CO_2_ gas experienced a more pronounced prevention of decreased body temperature compared to the group receiving regular CO_2_ gas [14], and the introduction of 21°C CO_2_ gas during laparoscopic surgery resulted in a temperature decrease to 35.2°C three hours post-surgery [13]. Previous studies have shown that the maintenance of normal body temperature was not achieved through skin warming, fluid warming, and humidification of anesthetic gases during laparoscopic surgery in elderly patients [14]. Therefore, it is necessary to further verify the effectiveness of maintaining normal body temperature with insufflation of CO_2_ gas warmed to 37°C. This may also be related to the duration of the dwell time after insufflation of CO_2_ gas, as the average dwell time of the experimental group in this study was 218 minutes, compared to 105 minutes in the study by Cheong [11], which is different from this study. Therefore, considering these findings, it can be inferred that intraperitoneal CO_2_ gas insufflation during laparoscopic surgery leads to intraoperative hypothermia. Warming interventions for temperature regulation are fundamentally important nursing interventions. Based on the results of this study, we believe that insufflation of warmed CO_2_ gas during laparoscopic surgery not only minimizes the decrease in intraoperative body temperature but also has the potential to be a nursing intervention to help subjects return to normal body temperature quickly in the recovery room after anesthesia has worn off. In this study, warmed CO_2_ gas was used as an insufflation agent in laparoscopic surgery and was found to affect acid-base balance, which is consistent with the results of a previous study conducted on animals using CO_2_ gas [20]. Based on Fick's diffusion law [20], which states that the diffusion coefficient of a substance is proportional to its temperature, Swenson et al. [21] support the theory by stating that warming carbon dioxide to 37°C increases carbon dioxide absorption through the peritoneum, resulting in decreased hemodynamic function and acid-base imbalance. These findings are similar to those of studies that have shown acid-base imbalance due to decreased body temperature during surgery [22]. Based on the above results, it is likely that acid-base imbalance is caused by a decrease in body temperature as the surgery time increases. These results suggest that warmed CO_2_ gas may affect acid-base balance, which may provide scientific support for future nursing interventions in postoperative hypothermia. In this study, warm insufflation of warmed CO_2_ gas was shown to affect lymphocytes. Previous studies [9] have shown that the use of insufflation of CO_2_ gas in laparoscopic surgery does not affect hemodynamic function and acid-base balance in healthy adults without high-risk factors such as heart and lung disease, but considering that the subjects in this study were elderly subjects aged 65 years or older, the results may be different due to age differences. In addition, the lymphocyte count in the group with warmed CO_2_ gas to 37°C was somewhat lower than that in the group with 21°C CO_2_ gas at 60 minutes after warming. However, the study showed that normal lymphocyte balance was restored after the end of surgery and anesthesia, indicating the need for further research to verify these findings in elderly patients. Tochihara et al. [15] reported that hypothermia is more likely to occur in the elderly than in other age groups due to imperfect vasoconstrictor mechanisms due to decreased elasticity with increasing age, and variability in thermoregulation due to decreased sympathetic nervous system reactivity and decreased metabolic heat production. Given that the subjects in this study were also elderly patients undergoing laparoscopic surgery, it is conceivable that there may be more age-related differences in body temperature than in other previous studies in adults. However, the subjects in this study were elderly subjects aged 65 years or older, and despite their reduced ability to regulate body temperature compared to adults, their intraoperative temperature decreased less, and their recovery to normal body temperature was rapid after surgery, and we believe that these results provide evidence that warm insufflation of CO_2_ gas is effective in preventing hypothermia and maintaining normal body temperature during surgery. As this study recruited subjects from a single center and the sample size was small, it is recommended that the number of subjects be expanded to a multicenter study to increase the representativeness of the sample and revalidate the effectiveness of warm insufflation of CO_2_ gas. In addition, the study controlled for the type of anesthetic and the duration of the surgery to confirm the effectiveness of warm insufflation of CO_2_ gas, but did not control for the effect of the experimental environment, which does not control for all factors that can increase body temperature. Therefore. we recommend further studies to control for exogenous variables that may increase body temperature and to determine changes in body temperature, acid-base balance, and lymphocytes in response to the warming effect of CO_2_ gas insufflation.

## CONCLUSION

This randomized controlled pre-post experimental study investigated the effects of intraperitoneal CO_2_ gas warming nursing care on body temperature, acid-base balance, and lymphocyte levels in elderly patients undergoing laparoscopic surgery. The results of this studysuggest that intraoperative application of warm insufflation of CO_2_ gas is an effective evidence-based nursing intervention for improving body temperature, acid-base balance, as well as lymphocyte balance in elderly laparoscopic surgery patients. This study provides nursing significance as it demonstrates the preventive effect against intraoperative hypothermia caused by general anesthesia and the stabilization of physiological indicators. By presenting theoretical evidence, it lays the foundation for practical nursing interventions applicable in clinical practice. In addition, it is hoped that in the future, insufflation of warmed CO_2_ gas will be used as a foundation for basic nursing theory and practice education for elderly patients undergoing laparoscopic surgery as an essential intraoperative nursing intervention.

## Figures and Tables

**Table 1.** Homogeneity Test of General and Disease-related Characteristics (N = 55)

Variables	Categories	Exp.	Cont.	t /F	χ^2^	*p*	Range
		(n = 29)	(n = 26)				
Age (years)		74.37 ± 6.35	74.03 ± 6.20	0.78		.420	65~87
Height (cm)		158.08 ± 8.62	160.13 ± 7.11	1.02		.092	135~177
Weight (kg)		61.35 ± 11.24	60.07 ± 10.75	1.20		.481	34~84
Diagnosis	Colon cancer	22 (75.9)	20 (76.9)	1.85		.420	
	Sigmoid cancer	1 (3.4)	1 (3.8)				
	Rectal cancer	6 (20.7)	5 (19.3)				
Sex	Men	17 (58.6)	16 (61.5)		1.11	.485	
	Women	12 (41.4)	10 (38.5)				
Anesthetics	Sevoflurane	25 (86.2)	21 (80.8)		2.02	.302^†^	
	Desflurane	4 (13.8)	5 (19.2)				
Operation	Anterior resection	10 (36.0)	10 (38.4)		1.93	.299	
	Hemicolectomy	12 (40.0)	10 (38.4)				
	Abdominoperineal resection	7 (24.0)	6 (23.2)				
Operating time (minutes)		171.45 ± 43.10	177.70 ± 52.98		1.42	.101	100~325
Anesthetic time (minutes)		212.21 ± 46.19	218.15 ± 30.08		0.87	.421	140~400

Values are presented as the mean ± standard deviation or n (%). Exp. = Experimental group; Cont. = Control group.

† Fisher exact test.

**Table 2.** Homogeneity Test of Dependent Variables in the Experimental and Control Groups (N = 55)

Variables	Exp.	Cont.	t	*p*
	(n = 29)	(n = 26)		
	M ± SD			
Baseline body temperature (°C)	36.77 ± 0.32	36.65 ± 0.28	1.01	.977
Baseline pH	7.46 ± 0.05	7.44 ± 0.01	0.59	.601
Baseline lymphocytes (%)	28.37 ± 9.49	25.68 ± 8.16	1.42	.201

Exp.= Experimental group; Cont. = Control group; M = Mean; SD = Standard deviation.

**Table 3.** Comparison of Body Temperature, Acid-base Balance, and Lymphocytes (N = 55)

Variables	Categories	Exp.	Cont.	Between groups		Source	F	*p*
		(n = 29)	(n = 26)					
		M ± SD		t / U	*p*			
Body temperature (°C)	T0	36.77 ± 0.32	36.65 ± 0.28	−0.45	.316	Group	0.85	.359
	T1	36.30 ± 0.36	36.27 ± 0.26	−0.45	.316	Time	9.49	< .001
	T2	36.07 ± 0.36	36.04 ± 0.30	2.12	.009	G*T	2.47	.010
	T3	35.83 ± 0.46	35.72 ± 0.34	2.28	.008			
Acid-base balance (pH)	T0	7.46 ± 0.05	7.44 ± 0.01	0.69	.493	Group	0.10	.751
	T1	7.39 ± 0.60	7.38 ± 0.04	2.91	.013	Time	4.95	< .001
	T2	7.37 ± 0.06	7.37 ± 0.05	4.26	< .001	G*T	2.39	.015
	T3	7.38 ± 0.05	7.38 ± 0.05	5.00	< .001			
Lymphocytes (%)	T0	28.37 ± 9.49	25.68 ± 8.16	0.53	.600	Group	1.64	.206
	T1	11.92 ± 14.36	9.90 ± 4.61	0.68	.394	Time	8.49	< .001
	T2	16.14 ± 14.53	13.00 ± 5.79	3.14	.011	G*T	4.45	.002
	T3	19.14 ± 12.92	14.86 ± 6.14	4.35	< .001			

Exp. = Experimental group; Cont. = Control group; M = Mean; SD = Standard deviation; G* T: Group*Time; T0 = Time before induction; T1 = 60 min after pneumoperitoneum; T2 = 120 min after pneumoperitoneum; T3 =180 min after pneumoperitoneum.

## Data Availability

The data that support the findings of this study are available from the corresponding author upon reasonable request.

## References

[1] Ministry of Health and Welfare, Korea Central Cancer Registry, National Cancer Center (2021). Annual report of cancer statistics in Korea in 2021 [Internet]. https://www.mohw.go.kr/board.es?mid=a10411010100&bid=0019&act=view&list_no=1482495&tag=&nPage=1.

[2] Korea Central Cancer Registry (2022). The 5-year survival rate for cancer patients is 72.9%, and 5% of the total population (approximately 2.59 million individuals) are cancer survivors [Internet]. https://www.mohw.go.kr/board.es?mid=a10503010100&bid=0027&act=view&list_no=1484074&tag=&nPage=1.

[3] National Cancer Information Center (2022). Site-specific cancer incidence statistics [Internet]. https://www.cancer.go.kr/lay1/S1T639C641/contents.do.

[4] Jiang R, Sun Y, Wang H, Liang M, Xie X (2019). Effect of different carbon dioxide (CO_2_) insufflation for laparoscopic colorectal surgery in elderly patients: a randomized controlled trial. Medicine.

[5] Giglia MD, Stein SL (2019). Overlooked long-term complications of colorectal surgery. Clinics in Colon and Rectal Surgery.

[6] Salibasic M, Pusina S, Bicakcic E, Pasic A, Gavric I, Kulovic E (2019). Colorectal cancer surgical treatment, our experience. Medical Archives.

[7] Zhong G, Abbas A, Jones J, Kong S, McCulloch T (2020). Environmental and economic impact of using increased fresh gas flow to reduce carbon dioxide absorbent consumption in the absence of inhalational anesthetics. British Journal of Anesthesia.

[8] Ko JC (2018). Small animal anesthesia and pain management.

[9] Gunusen I, Akdemir A, Sargın A, Karaman S (2022). The effects of CO_2_ pneumoperitoneum at different temperature and humidity on hemodynamic and respiratory parameters and postoperative pain in gynecological laparoscopic surgery: a prospective randomized controlled study. Asian Journal of Surgery.

[10] Ghaffari‐Bohlouli P, Jafari H, Okoro OV, Alimoradi H, Nie L, Jiang G (2024). Gas therapy: generating, delivery, and biomedical applications. Small Methods,.

[11] Cheong JY, Keshava A, Witting P, Young CJ (2018). Effects of intraoperative insufflation with warmed, humidified CO_2_ during abdominal surgery: a review. Annals of Coloproctology.

[12] Tian YN, Gao WY, Tian XR, Wang ZW (2022). Comparative efficacy of six active warming systems for intraoperative warming in adult patients undergoing laparoscopic surgery: a systematic review and network meta-analysis. Therapeutic Hypothermia and Temperature Management.

[13] Kordestani SV, Nasiri-Formi E, Qane MD, Khodabakhsh E (2025). Effects of warm carbon dioxide insufflation vs. local heat on shoulder pain in laparoscopic cholecystectomy: a randomized clinical trial. Journal of Thermal Biology.

[14] Park HS, Shin NY (2019). Physiological factor evaluation of the warm humidification of anesthetic gas nursing care. Asia-Pacific Journal of Oncology Nursing.

[15] Tochihara Y, Yamashita K, Fujii K, Kaji Y, Wakabayashi H, Kitahara H (2021). Thermoregulatory and cardiovascular responses in the elderly towards a broad range of gradual air temperature changes. Journal of Thermal Biology.

[16] Haynes SR, Lawler PG (1995). An assessment of the consistency of ASA physical status classification allocation. Anesthesia.

[17] Kim DS (2008). Basics Q & A for anesthesia residents.

[18] Park HS, Kang YH (2018). Effects of heated-humidified anesthetic gas in the elderly patients with colorectal cancer during laparoscopic surgery: randomized controlled trial. Korean Journal of Adult Nursing.

[19] Morris RH, Wilkey BR (1970). The effect of ambient temperature on patient temperature during surgery not involving body cavities. Anesthesiology.

[20] Bashirov E, Cetiner S, Emre M, Seydaliyeva T, Alic V, Daglioglu K et al (2007). A randomized controlled study evaluating the effects of the temperature of insufflated CO_2_ on core body temperature and blood gases (an experimental study). Surgical Endoscopy.

[21] Swenson KE, Swenson ER (2021). Pathophysiology of acute respiratory distress syndrome and COVID-19 lung injury. Critical Care Clinics.

[22] Park JI, Yoon HS (2017). Effects of 37˚C carbon dioxide pneumoperitoneum on core body temperature, systolic blood pressure, heart rate and acid-base balance: a randomized double-blind controlled trial. Journal of Korean Biological Nursing Science.

